# Lateralized interactive social content and valence processing within the human amygdala

**DOI:** 10.3389/fnhum.2012.00358

**Published:** 2013-01-18

**Authors:** Pascal Vrtička, David Sander, Patrik Vuilleumier

**Affiliations:** ^1^Center for Interdisciplinary Brain Sciences Research, Department of Psychiatry and Behavioral Sciences, School of Medicine, Stanford UniversityStanford, CA, USA; ^2^Swiss Center for Affective Sciences, University of GenevaGeneva, Switzerland; ^3^Laboratory for Neurology and Imaging of Cognition, Department of Neurology and Department of Neurosciences, University Hospital and Medical School, University of GenevaGeneva, Switzerland; ^4^Laboratory for the Study of Emotion Elicitation and Expression (E3 Lab), Department of Psychology, FPSE, University of GenevaGeneva, Switzerland

**Keywords:** social content, valence, human amygdala, trait anxiety, fMRI

## Abstract

In the past, the amygdala has generally been conceptualized as a fear-processing module. Recently, however, it has been proposed to respond to all stimuli that are relevant with respect to the current needs, goals, and values of an individual. This raises the question of whether the human amygdala may differentiate between separate kinds of relevance. A distinction between emotional (vs. neutral) and social (vs. non-social) relevance is supported by previous studies showing that the human amygdala preferentially responds to both emotionally and socially significant information, and these factors might even display interactive encoding properties. However, no investigation has yet probed a full 2 (positive vs. negative valence) × 2 (social vs. non-social content) processing pattern, with neutral images as an additional baseline. Applying such an extended orthogonal factorial design, our fMRI study demonstrates that the human amygdala is (1) more strongly activated for neutral social vs. non-social information, (2) activated at a similar level when viewing social positive or negative images, but (3) displays a valence effect (negative vs. positive) for non-social images. In addition, this encoding pattern is not influenced by cognitive or behavioral emotion regulation mechanisms, and displays a hemispheric lateralization with more pronounced effects on the right side. Finally, the same valence × social content interaction was found in three additional cortical regions, namely the right fusiform gyrus, right anterior superior temporal gyrus, and medial orbitofrontal cortex. Overall, these findings suggest that valence and social content processing represent distinct kinds of relevance that interact within the human amygdala as well as in a more extensive cortical network, likely subserving a key role in relevance detection.

## Introduction

Thanks to the advancement of neuroimaging techniques and paradigms, our knowledge on human amygdala function has steadily increased during the last two decades. Yet, divergent views have emerged concerning stimulus properties that trigger amygdala responses. One classic notion is that the amygdala constitutes a *fear module* crucially involved in the automatic detection of threat-related information, fear reaction and fear learning (Ohman and Mineka, 2001). Other views suggest instead that the amygdala may preferentially process arousal (Anderson et al., 2003; Small et al., 2003) *or* valence (Murray, 2007) information. According to such notions, the amygdala would either represent a general *arousal indicator* or * valence processor*. Finally, besides these traditional accounts respectively derived from basic (Ekman, 1999) or bi-dimensional (Russell, 1980) theories of emotion, recent experimental evidence revealed amygdala activation to be determined by many additional factors (not confounded by arousal), such as eye gaze (N'Diaye et al., 2009; Sato et al., 2010), novelty (Blackford et al., 2010; Weierich et al., 2010; Balderston et al., 2011), social content (Norris et al., 2004; Britton et al., 2006; Goossens et al., 2009; Scharpf et al., 2010), context (Kim et al., 2003, 2004; Vrticka et al., 2008), personal impact (Ewbank et al., 2009), or individual differences in subjective evaluation (Schiller et al., 2009), motivational state (Canli et al., 2001; LaBar et al., 2001; Morris and Dolan, 2001) as well as various psychological traits (Canli et al., 2001; Bishop et al., 2004; Etkin et al., 2004; Sabatinelli et al., 2005; Dickie and Armony, 2008; Vrticka et al., 2008, 2012a; Vrticka and Vuilleumier, 2012). To integrate these different findings, a new account of human amygdala function has been put forward, primarily linking it with the appraisal of biological relevance (Sander et al., 2003; Sergerie et al., 2006; Adolphs, 2010; Pessoa and Adolphs, 2010). This concept of relevance detection has its origins in emotion psychology (Sander et al., 2003), in particular in appraisal theories of emotion (Sander et al., 2005), and refers to the preferential processing of events that are (biologically) relevant to major concerns/goals/needs and values of an individual at a specific moment in time (see Frijda, 2009; Reisenzein, 2009).

When considering human amygdala function in terms of biological relevance detection, the question arises what kind of information might be most relevant, and thus which stimulus properties are preferentially processed by the human amygdala. To address this issue, several studies have compared two different kinds of relevance, in particular emotional *vs.* social relevance (Norris et al., 2004; Britton et al., 2006; Harvey et al., 2007; Scharpf et al., 2010). On the one hand, *emotional relevance* was referred as to stimuli that are likely to be appraised so that they would elicit an emotional response (see Sander et al., 2005) and modulate cognitive processes such as attention (Vuilleumier, 2005; Brosch et al., 2008), independently of their social nature. On the other hand, *social relevance* was associated with stimuli conveying information about interpersonal interactions and conspecifics, regardless of their emotional value. This corroborates the view denoting a high relevance of social information for our species due to its direct link to guiding human behavior (Keltner and Kring, 1998; Hariri et al., 2002). It is also consistent with the so called social brain hypothesis (Dunbar, 1998), stating that the need for social skills led to functional specialization of new cognitive mechanisms and “*… fuelled the expansion of the human brain …*” (Adolphs, 2003, p. 166). Accordingly, the abovementioned investigations reported both emotional relevance effects (emotional vs. neutral) and social relevance effects (social vs. non-social) in the amygdala (Norris et al., 2004; Britton et al., 2006; Scharpf et al., 2010). The last study even suggests an interactive processing of emotional and social relevance (highest amygdala activation for emotional *and* social stimuli), although no formal test for such interactive processing was carried out (Scharpf et al., 2010). Such data corroborate the notion that socially relevant stimuli are most likely also emotionally relevant, in the sense that emotions are typically elicited in social situations or by taking into account the affective or motivational dimension of social contexts (Jakobs et al., 1997; Balderston et al., 2011). However, none of these studies differentiated the emotional relevance effect in terms of valence, distinguishing between positive *vs.* negative value (Morrison and Salzman, 2010), therefore leaving the question open of whether valence processing (e.g., threat) may (at least partly) determine the response to social relevance. According to the literature, negative stimuli should activate the amygdala stronger than positive ones due to their intrinsically higher biological relevance in terms of survival (Hariri et al., 2002). To clarify relevance detection in the human amygdala, it would therefore be useful to test for a full 2 (valence) × 2 (social content) interaction pattern, which was not possible in previous studies (Norris et al., 2004; Britton et al., 2006; Harvey et al., 2007; Scharpf et al., 2010). Consequently, the current functional magnetic resonance imaging (fMRI) study applied such experimental design, using both social and non-social *neutral* images serving as an additional baseline.

In addition, fMRI investigations in the field of emotion regulation have disclosed differential amygdala activation to positive and negative stimuli as a function of task instructions or viewing conditions, particularly through emotion regulation processes. The latter may involve cognitive (re-appraisal) or behavioral (expressive suppression) regulation strategies, aimed at either up- or down-regulating emotional states (Ochsner et al., 2002, 2004; Levesque et al., 2003; Kim and Hamann, 2007; Goldin et al., 2008). We therefore also included these two major regulation strategies (together with a natural viewing condition) in our experimental paradigm to further test for potential task effect on relevance detection mechanisms.

Furthermore, as already mentioned above, amygdala activity is consistently modulated as a function of motivational state (Canli et al., 2001; LaBar et al., 2001; Morris and Dolan, 2001) or personality traits (Canli et al., 2001; Bishop et al., 2004; Sabatinelli et al., 2005; Vrticka et al., 2008, 2012a,b; Vrticka and Vuilleumier, 2012). Therefore, we also included measures assessing individual differences in the current study, in order to probe for relations between relevance detection mechanisms and personal dispositions. First, we chose trait anxiety (STAI-T, see section “Methods”), because it has already previously been shown to modulate amygdala activity to threat- or fear-related stimuli (Bishop et al., 2004; Etkin et al., 2004; Dickie and Armony, 2008), and thus influence negative valence processing. Second, we assessed attachment style (secure vs. insecure—avoidant or anxious; Relationships Scales Questionnaire, see section “Methods”), because we have previously shown that this personality trait can influence social emotional processing within the human amygdala and increase blood-oxygen-level-dependent (BOLD) responses to social negative interaction scenarios (Vrticka et al., 2008, 2012a; Vrticka and Vuilleumier, 2012).

Finally, besides the question to what stimulus types or properties the amygdala would respond to preferentially, another issue on human amygdala activation concerns any *hemispheric lateralization* during social and/or emotional processing. Hemispheric lateralization of emotion in general has been proposed in different ways, either in terms of (1) fundamentally more right-lateralized emotion processing, regardless of valence; (2) a preferential representation of positive vs. negative emotions in the left vs. right hemispheres, respectively—and thus as a function of valence; or (3) a distinction between approach (left) vs. avoidance (right) behavioral tendencies, rather than valence (Sergerie et al., 2006). Regarding lateralization in the human amygdala more specifically, one prominent account has been related to language and proposed a differential representation of semantic (left) vs. non-semantic (right) information (Sergerie et al., 2006), whereas another hypothesis highlighted differential temporal dynamics and suggested faster emotional information processing with quicker habituation in the right as compared to the left amygdala (Sergerie et al., 2006). Amygdala lateralization has also been related to sex differences (Cahill, 2006), but here we chose to recruit only female participants to avoid confound related to this additional factor. However, assumptions on hemispheric lateralization have generally focused on the valence dimension of emotional information only, and it still remains to be seen how such lateralization accounts may be affected by social relevance.

According to the previous literature and theoretical considerations of the appraisal theory summarized above (Sander et al., 2003), we predicted the following findings. First, the amygdala should display a social *vs.* non-social activation difference (for both neutral and emotional stimuli), because social information is thought to be particularly relevant for our species. Second, this social relevance effect should interact with the representation of valence in terms of a negative *vs.* positive activation difference, as social threat is likely to be even more strongly relevant. Third, personality (trait anxiety and/or attachment style) should modulate these valence and social effects in the human amygdala, because the appraisal of social emotional stimuli is strongly dependent on their significance for the observer in terms of personal values and needs. Thereby, social *and* emotional stimuli should be affected most, again due to their highest significance for the human species.

## Methods

### Subjects

We recruited 19 healthy paid volunteers (all right-handed women, mean age 24.82 ± 4.0), who all had a normal or corrected to normal vision, no history of neurological or psychiatric disease, and gave informed written consent according to the local ethical committee regulation. Only women were included in order to avoid any potential sex differences that could have modulated the effects of interest (see e.g., Cahill, 2006; Kim and Hamann, 2007; Vrticka et al., 2012a,b). fMRI data from the same study were previously reported in Vrticka et al. (2011) and Vrticka et al. (2012a), but the latter focused on whole-brain results comparing specific emotion regulation strategies and distinct attachment styles. In addition, behavioral data derived from stimulus validation (see section “Stimuli” below) was published in Vrticka et al. (2012b). Here, we specifically examine amygdala activity and brain areas that show main effects of social and emotional relevance, independently of any task effects, and we investigate the role of trait anxiety (rather than individual attachment style, see below).

### Experimental material and procedure

#### Stimuli

A total number of 360 emotional pictures were initially collected from the internet and from the International Affective Pictures System (IAPS). All were in colors, and adjusted to obtain similar size, contrast, and pixel resolution. Half of the pictures displayed scenes with a clear social content, such as two people fighting or a mother interacting with her baby. The other half represented animals, objects or landscapes that were not social, like a dead bird in industrial waste or a tropical island scene. All 360 pictures were rated in a separate behavioral study by 54 female students on three continuous rating scales (from 1 to 100), including PLEASANTNESS (from very negative to very positive), INTENSITY (from low to high arousal), and CONTROL (from absence to full presence of control over the emotional experience induced by viewing images (Vrticka et al., 2012b). According to the average rating results from this sample, 240 pictures were finally chosen for the fMRI study, and sorted by their SCENE CONTENT (either social or non-social) and VALENCE (either positive or negative). This gave rise to four stimulus categories (60 pictures each): Social Positive (SP) or Negative (SN), and Non-social Positive (NSP) or Negative (NSN). Negative images were rated as lower in pleasantness and control, but higher in intensity than positive images (*ps* < 0.001). However, there were no differences between social vs. non-social images (*ps* > 0.25), and no interactions (*ps* > 0.11). In addition, there were no differences in luminance overall (*ps* > 0.098), and social complexity for social images specifically (number of humans per image; *p* = 0.5). Please refer to Vrticka et al., (2011 and 2012a,b) for detailed values. Note that the differences in intensity between negative and positive stimuli could not be avoided in order to match pairs of social and non-social scenes in both valence conditions, because social material is otherwise typically judged as much more intense than non-social material (Ewbank et al., 2009). Finally, we also selected 40 neutral images from the IAPS database (20 including humans, 20 without humans) to be used in a baseline control condition (see below), with valence ratings situated between positive and negative images.

#### Experimental conditions

Before entering the fMRI scanner, all participants were told that the purpose of the experiment would be to investigate how the brain reacts to different types of images (e.g., real scenes *vs.* fiction scenes) and to which degree people can voluntarily influence the emotional impact of these images on them. Accordingly, the experimental layout comprised four different viewing conditions in which pictures were presented with different tasks to induce different emotion regulation strategies.

The first condition was used as a control baseline, and was introduced to the participants as “a photographic quality” judgment, where they had to indicate on each trial (by button press, using a 4-point scale—see below) whether the image was of good quality (e.g., well-focused or properly lighted). All images in this condition were neutral, but could display either scenes with humans (i.e., social content) or inanimate settings and landscapes (i.e., non-social content). This viewing condition was later used to provide a baseline for general differences in brain activation to social *vs.* non-social stimuli, irrespective of emotional processing demands and valence. It was presented as the first block of the first scanning run and the last block of the last run.

The three other viewing conditions included emotional images only, and comprised an emotion experience, a cognitive re-evaluation (re-appraisal), and a behavioral expressive suppression condition. Because there was no significant three-way interaction in any region of interest (see section “Results”), activations were collapsed during final data analysis. For more details regarding the experimental layout, please refer to the study where the emotion regulation effects were explored systematically as a function of the different viewing conditions (Vrticka et al., 2011).

The participant's task was to report their feeling state evoked by the preceding stimulus (“How did you feel while seeing the last image”?), using a 4-point scale (see below). All emotional images were counterbalanced across participants, so that the same images seen in one viewing condition by a given participant were seen in the other viewing conditions by different participants.

#### Procedure

The fMRI experiment was divided into three successive scanning runs. Each run included two of the three viewing conditions, presented in blocks of 40 emotional images (duration = 294 s per block), whereas the first and the last run also included an additional block of 20 neutral images (baseline condition, duration = 151 s). Within each block, images were pseudo-randomized and equally probable for the different stimulus categories (social *vs.* non-social content, positive *vs.* negative valence). The first and the third runs lasted approximately 13 min, and the middle run 10 min.

**Figure 1:**
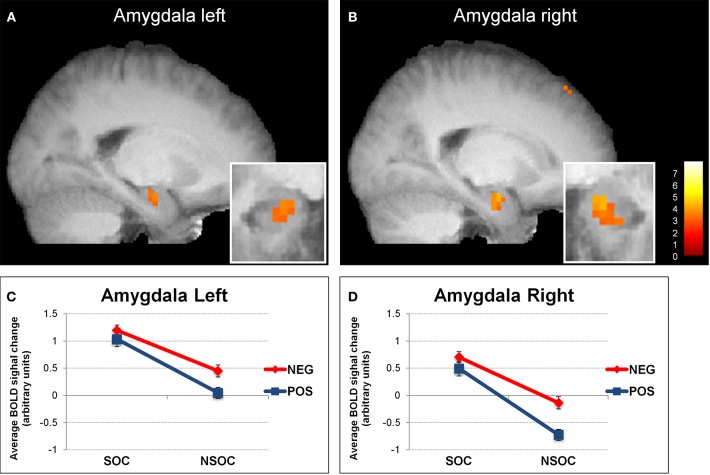
**Interactive valence and social content processing in bilateral amygdala.** Statistical parametric maps (threshold *p* = 0.005) for the contrast social > non-social displaying increased activity in the left **(A)** and right **(B)** amygdala, (big windows: sagittal view; small windows: coronal view). Graphs depicting the extracted activation values (betas) averaged across voxels from the left **(C)** and right **(D)** amygdala, displaying a valence × social content interaction. NEG, negative; NSOC, non-social; POS, positive; SOC, social. Error bars reflect S.E.M.

Each viewing condition block began with an instruction display (7 s), followed by images in pseudo-randomized order. Every individual trial started with a fixation cross at the screen center (average duration = 1125 ms jittered between 790 and 1485 ms), followed by an emotional or neutral image for 2 s, and then a response display probing for emotion ratings (4 s; see Figure 1). Ratings were made on a 4-button response box, according to a 4-point scale ranging from very and slightly negative (buttons 1 and 2, respectively) to slightly and very positive (buttons 3 and 4, respectively).

#### MRI acquisition

MRI data were acquired on a 3 T whole-body scanner (Siemens TIM TRIO, Erlangen, Germany), using standard head-coil configuration. For each participant, a structural image was obtained with a MPRAGE T1-weighted sequence (TI/TR/TE/flip = 900/1900/2.32/9°, parallel acquisition (GRAPPA) with acceleration factor 2, FOV = 230 × 230 × 173 mm^3^, Matrix = 256 × 246 × 192). Functional images (TR/TE/Flip = 2200 ms/30 ms/85°, parallel acquisition (GRAPPA) with acceleration factor 2, FOV = 235 mm × 235 mm, matrix = 128 × 84, resulting voxel size is 2.8 × 1.8 × 3.4 mm^3^) covered the whole brain, composed of 36 contiguous 4 mm axial slices parallel to the inferior edge of the occipital and temporal lobes, and acquired continuously for a total of 975 images per participant (two sessions with 350 and one session with 275 images).

Image processing was performed with SPM2 (www.fil.ion.ucl.ac.uk) using standard procedures for realignment of the time-series, slice-timing correction, normalization to a standard brain template in MNI space, and smoothing with an 8 mm FWHM Gaussian kernel. Statistical analysis was performed using the general linear model implemented in SPM2, with a separate regressor for each event type convolved with a canonical hemodynamic response function. Twelve event types from the emotion regulation task (4 image categories: SP, NSP, SN, and NSN; for each of the three viewing conditions), plus two additional event types (social and non-social) from the baseline condition were modeled for each participant, using the three scanning runs in a fixed-effect analysis at the single-subject level. Movement parameters from realignment corrections were entered as additional covariates of no interest for each scanning run, in order to account for residual movement artifacts after realignment. Statistical parametric maps were then generated from linear contrasts between the different conditions in each participant.

A second-stage random-effect analysis was performed using one-sample *t*-tests on contrast images obtained in each subject for each comparison of interest. Because of *a-priori* predictions regarding amygdala, we opted to threshold amygdala activations at *p* < 0.005 (uncorrected, whole brain) and *k* ≥ 10. All other contrasts were thresholded at *p* < 0.001 (uncorrected, whole brain) and *k* ≥ 20 (Lieberman and Cunningham, 2009). Average parameter estimates of activity (betas) for each condition were extracted from all voxels in regions of interest, defined by the full-extent clusters showing significant activation at a voxel level in the SPM group analysis (random-effect contrasts). These beta values were then used for repeated-measure ANOVAs, ANCOVAs and *t*-tests performed in SPSS (Version 18; http://www.spss.com/) with the factors of social content, valence, viewing condition, and personality, when appropriate.

#### Individual diffrence measures

Trait anxiety was measured using a French version of the Spielberger State-Trait Anxiety Inventory (STAI-T) analyzed according to the author's manual (Spielberger, 1983). Attachment style was assessed by a validated French version (Guédeney et al., 2010) of the relation scales questionnaire (RSQ; Collins and Read, 1994), analyzed according to Kurdek (Kurdek, 2002) relying on an initial model by Simpson and colleagues (Simpson, 1990; Simpson et al., 2002)—see also (Vrticka et al., 2008, 2012a,b).

Influences of trait anxiety or attachment style on brain activity in regions of interest were assessed by a full 3 (viewing condition) × 2 (valence) × 2 (social content) × 1 (anxiety) or × 2 (attachment style) analysis of covariance (ANVCOVA), by including the respective personality measures for each subject in SPSS (www.ibm.com/software/analytics/spss/). To do so, the raw scores collected from STAI-T and attachment scales were centered to avoid multicollinearity problems in multiple regression (Aiken and West, 1991).

## Results

### Behavioral results

A full 3 (viewing condition) × 2 (valence) × 2 (social content) ANOVA on the behavioral ratings revealed (1) a main effect of valence [*F*_(1, 18)_ = 1149, *p* < 0.001] because positive images were always rated as more pleasant than negative images, and (2) a viewing condition × valence interaction [*F*_(1, 18)_ = 36.79, *p* < 0.001], because both positive and negative images were rated as less pleasant and less unpleasant, respectively, during re-appraisal as compared to emotion experience and expressive suppression (Vrticka et al., 2011).

### Main effects and interactions of the fMRI analysis

In an initial step, we computed the four main effects contrast regarding valence (positive > negative and vice versa) and social content (social > non-social and vice versa). These analyses revealed significant BOLD signal change in the amygdala solely for the contrast social > non-social (see Table 1 and Figure 1). Because the main aim of the present study was to investigate relevance detection within the amygdala (and only subsidiarily examine any additional brain areas displaying the same computational profile), all subsequent analysis steps are reported for this region first, and then for regions appearing in the same initial social > non-social comparison (functional region of interest determination; see Table 1).

**Table 1:** **Brain areas activated in the main contrast SOCIAL > NON-SOCIAL, listed with peak coordinates and best estimates of anatomical location**.

**Region**	**BA**	**Voxel**	***T*-Value**	***x***	***y***	***z***
**SOCIAL > NON-SOCIAL**				
Amygdala right^*^		26	4.21	21	−6	−18
Amygdala left^*^		13	3.55	−21	−9	−18
Fusiform Gyrus right	37	98	6.59	42	−42	−27
Anterior STG right	21	111	6.29	60	−6	−24
mOFC	11	40	5.26	−3	54	−18
mPFC	10	28	4.36	3	57	15
Temporal Inferior left	20/21	109	6.85	−57	−3	−27
PCC	23	393	7.32	0	−51	33
pSTS left	19	458	7.82	−45	−84	0
pSTS right	19	711	7.07	45	−48	18
Fusiform Gyrus left	19/37	25	4.43	−42	−63	−21
Occipital cortex left	17	331	7.74	−6	−102	9

Statistical threshold was p < 0.005 (uncorrected, whole brain) and k = 10 for bilateral amygdala as indicated by

* above, and p < 0.001 (uncorrected, whole brain) and k ≥ 20 for remaining brain areas. STG, superior temporal gyrus; OFC, orbitofrontal cortex; PFC, prefrontal cortex; STS, superior temporal sulcus; PCC, posterior cingulate cortex; m, medial; dl, dorsolateral; dm, dorsomedial; p, posterior. The first five brain areas listed were the ones found to display a significant valence × social content interaction; BA, brodman area.

#### Amygdala

We first analyzed amygdala activation during the neutral control condition with a 2 (side) × 2 (social content) ANOVA. This confirmed a main effect of social content [social > non-social; *F*_(1, 18)_ = 9.78, *p* = 0.006], demonstrating that this effect was present even for non-emotional scenes (see Figure 3A).

We then analyzed amygdala activation to emotional images during the three different viewing conditions with a 2 (side) × 3 (viewing instruction) × 2 (valence) × 2 (social content) ANOVA. This revealed several main effects and interactions (see Figure 1). First, there was a main effect of side [*F*_(1, 18)_ = 2699, *p* < 0.001], because activity was overall higher in the left than in the right amygdala. Second, we found a main effect of valence [*F*_(1, 18)_ = 7.37, *p* = 0.014], as activity was overall higher for negative as compared to positive images. There was also a side × valence interaction [*F*_(1, 18)_ = 7.15, *p* = 0.016], because the activation difference between negative *vs.* positive images was greater in the right [*t*_(18)_ = 2.73, *p* = 0.014] as compared to the left [*t*_(18)_ = 2.21, *p* = 0.04] amygdala. Third, our data showed a main effect of social content [*F*_(1, 18)_ = 38.39, *p* < 0.001], because activity was overall higher for social than non-social scenes.

We also found a side × social content interaction [*F*_(1, 18)_ = 6.25, *p* = 0.022], which arose because of a more pronounced social > non-social activation difference in the right [*t*_(18)_ = 5.83, *p* < 0.001] as compared to the left [*t*_(18)_ = 4.29, *p* < 0.001] amygdala. These effects were accompanied with a valence × social content [*F*_(1, 18)_ = 7.30, *p* = 0.015] and a marginally significant side × valence × social content [*F*_(1, 18)_ = 3.89, *p* = 0.064] interaction. The valence × social content interaction showed that activity in both amygdalae was not significantly different between negative and positive social images [all *t*_(18)_ < 1.51, *p* > 0.15], but higher for negative than positive non-social images [all *t*_(18)_ > 2.66, *p* < 0.016], while the side × valence × social content interaction revealed that this effect was stronger in the right [*t*_(18)_ = 10.24, *p* = 0.005] as compared to the left [*t*_(18)_ = 3.95, *p* = 0.062] amygdala.

Finally, there were no interactions with viewing conditions {natural viewing, re-appraisal, and suppression; [all *F*_(1, 18)_ < 1.12, *p* > 0.34]}, suggesting that the abovementioned valence and social relevance detection pattern was independent of task instructions.

Overall, these data on amygdala activation revealed two main findings. Firstly, we observed a reliable preferential response to social > non-social information bilaterally, for both neutral and emotional images. Secondly, we also found an additional interactive processing of valence and social content, which was more right-lateralized.

#### Other areas in social brain networks

**Figure 2:**
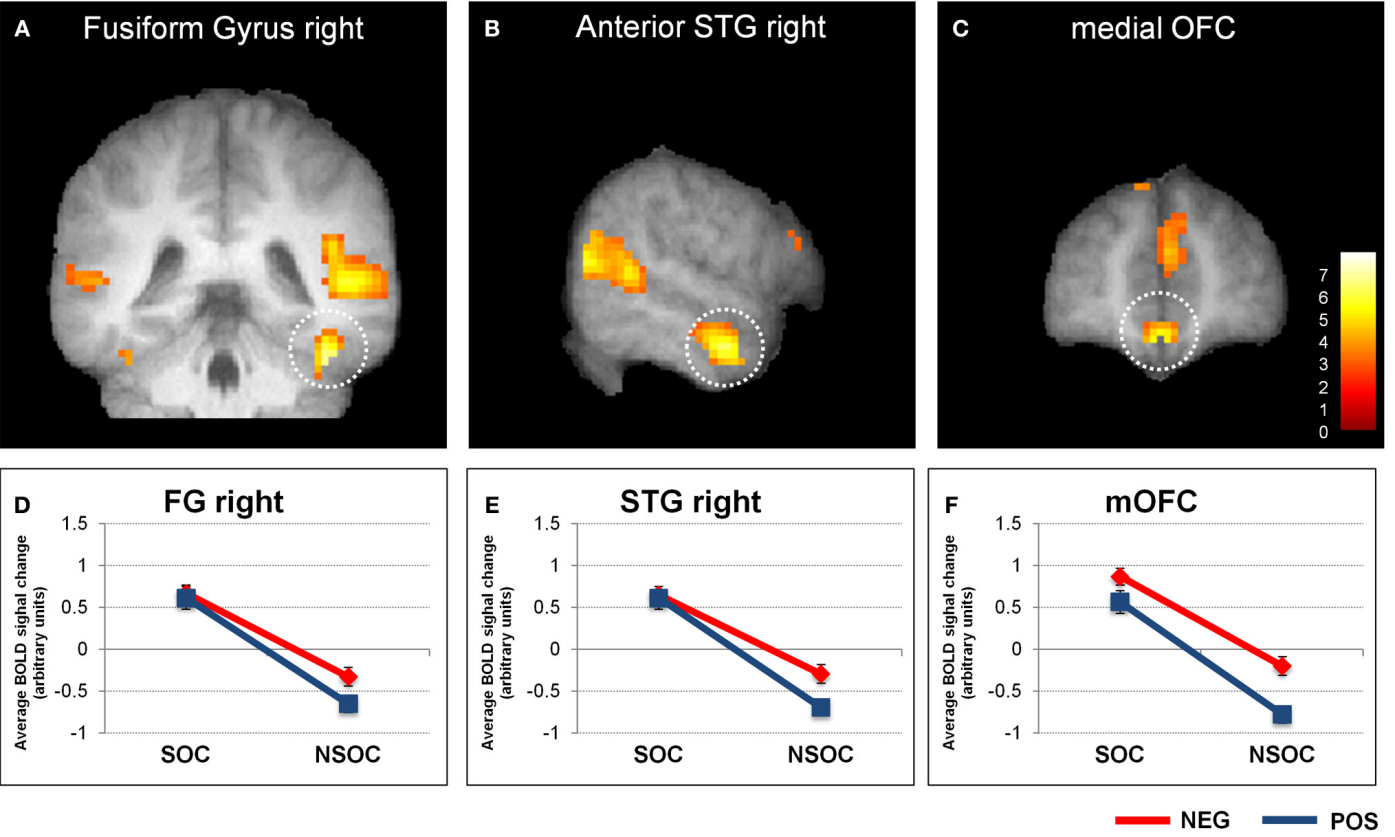
**Interactive valence and social content processing in fusiform gyrus, right anterior superior temporal gyrus, and medial orbitofrontal cortex.** Statistical parametric maps (threshold *p* = 0.001) for the contrast social > non-social displaying significant activity in the right fusiform gyrus **(A)**, right anterior superior temporal gyrus **(B)**, and medial orbitofrontal cortex **(C)**. Graphs depicting averaged extracted raw activation values (betas) in right fusiform gyrus **(D)**, right anterior superior temporal gyrus **(E)**, and medial orbitofrontal cortex **(F)**, displaying a valence × social content interaction. NEG, negative; NSOC, non-social; POS, positive; SOC, social. Error bars reflect S.E.M.

The 3 × 2 × 2 ANOVA was similarly performed on extracted beta values from the other regions of interest that were identified by the same initial computation of the social > non-social main effects (see Table 1). Like in the amygdala, this revealed a significant valence × social content interaction in the right fusiform gyrus (FG), right anterior superior frontal gyrus (SFG), and medial orbito-frontal cortex (mOFC; see Figure 2). In all these three brain areas, there were also main effects of valence [negative > positive; all *F*_(19, 1)_ > 5.67, *p* < 0.028] and social content [social > non-social; all *F*_(19, 1)_ > 65.63, *p* < 0.001], but no interaction with the three different viewing conditions {natural viewing, re-appraisal, suppression; [all *F*_(19, 1)_ < 2.39, *p* > 0.11]}.

**Figure 3:**
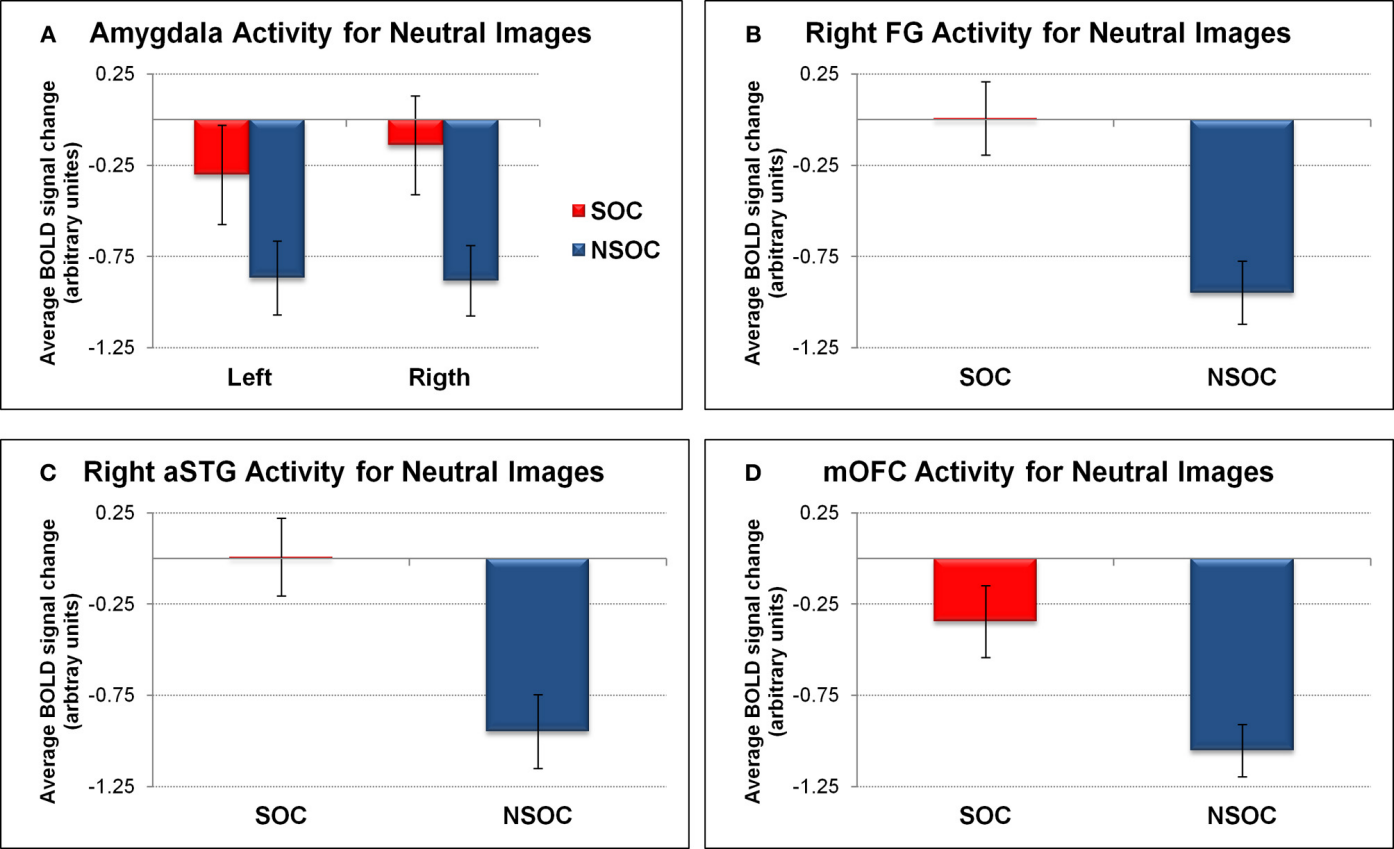
**Social relevance effect for neutral images in regions of interest.** Social > non-social activation difference for neutral images in **(A)** bilateral amygdala, **(B)** right fusiform gyrus, **(C)** right anterior superior temporal gyrus, and **(D)** medial orbitofrontal cortex. FG, fusiform gyrus; NSOC, non-social; mOFC, medial orbitofrontal cortex; SOC, social; aSTG, anterior superior temporal gyrus. Error bars reflect S.E.M.

We also assessed activity in the FG, aSTG, and mOFC during the neutral control condition. This revealed a significant social > non-social activation difference in all three regions [all *F*_(1, 18)_ > 16.03, all *p* = 0.001] (see Figures 3B,C,D).

The findings in the FG, aSTG, and mOFC thus showed a very similar pattern to the amygdala, with preferential social vs. non-social processing, regardless of emotional content, but also interactive processing of valence and social content, independent of viewing conditions.

### Correlations with personality measures

We next assessed the possible influence of trait anxiety and adult attachment style on relevance detection using regression analyses with the corresponding questionnaire scores (see section “Methods” for details).

#### Amygdala

**Figure 4:**
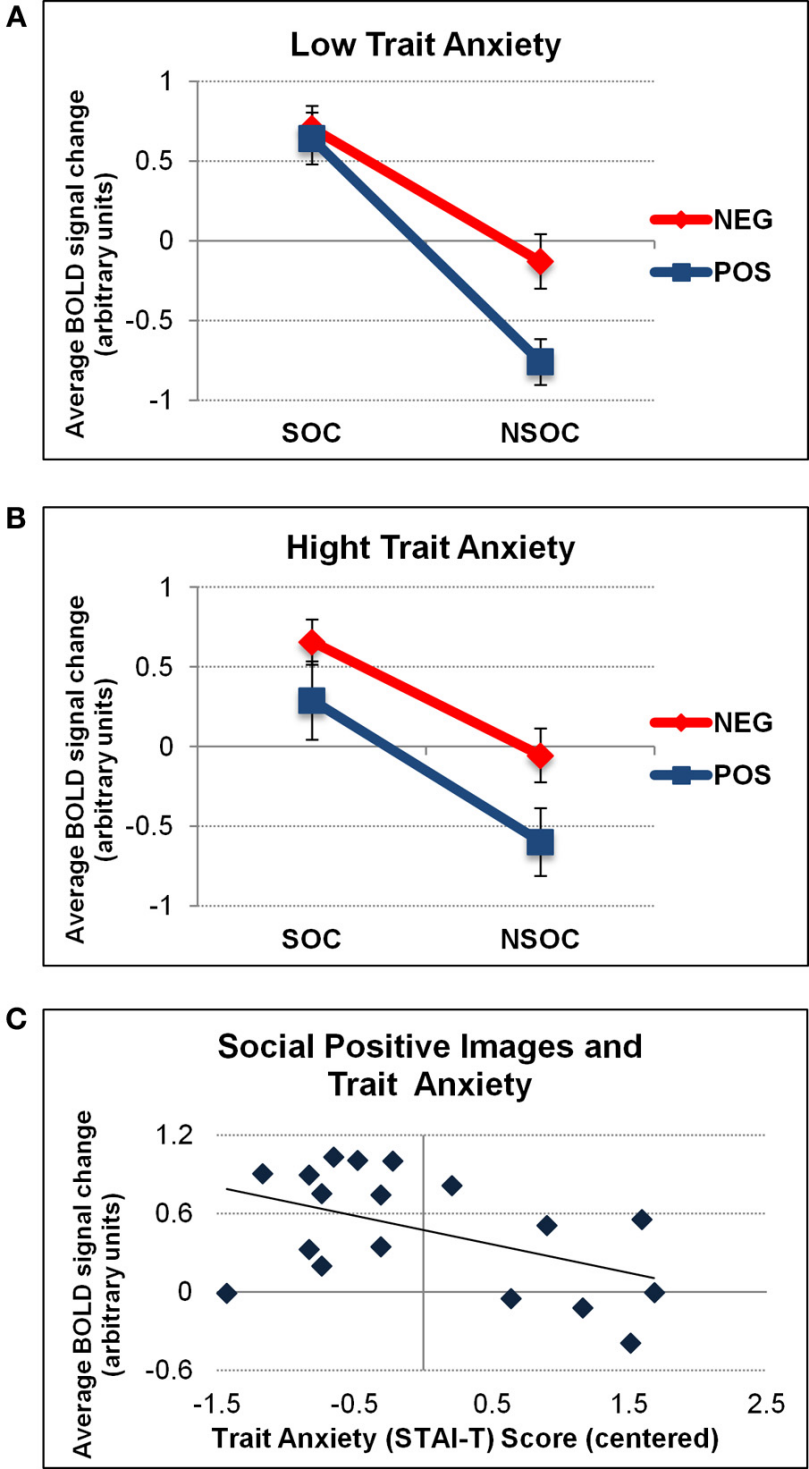
**Interactive valence and social content processing in right amygdala as a function of trait anxiety.** Graphs depicting the average activation values (betas) extracted from the right amygdala as a function of low **(A)** and high **(B)** trait anxiety (STAI-T). A significant valence × social content interaction was only found for low anxious individuals. The latter was due to a selective effect of trait anxiety on activation to social positive (SP) images **(C)**. NEG, negative; NSOC, non-social; POS, positive; SOC, social. Error bars reflect S.E.M.

When considering individual differences using trait anxiety scores in an ANCOVA, we found a trend for a 2 (valence) 2 × (social content) × 1 (trait anxiety) triple interaction in the right amygdala [*F*_(1, 18)_ = 4.19, *p* = 0.059]. There was no such effect in the left amygdala [*F*_(1, 18)_ = 1.63, *p* = 0.22], and there were no additional interactions with trait anxiety bilaterally. To visualize the direction of these effects, we performed a median split of activation parameters from the right amygdala according to individual trait anxiety scores (low: *n* = 8, mean AX score = −0.86; high: *n* = 8, mean AX score = 0.93). This visualization revealed that the right amygdala effect was explained by a significant valence × social content interaction solely for low [*F*_(1, 7)_ = 17.06, *p* = 0.004] but not high [*F*_(1, 7)_ = 0.68, *p* = 0.44] anxious subjects (see Figures 4A,B). To further elaborate on the finding that high anxious participants did not display any valence × social content interaction pattern in the right amygdala, we correlated activation values (betas) for each stimulus condition separately (SP, NSP, SN, NSN) with trait anxiety scores from all subjects. This revealed a selective negative association between trait anxiety and activation for social positive (SP) images (*r*^2^ = 0.24, *p* = 0.041), in the sense that the higher the trait anxiety, the lower the right amygdala activation to SP images (see Figure 4C). No such relations were found for the three other image categories (*r*^2^ < 0.05).

The valence × social content interaction for high anxious participants was unaffected by the different viewing conditions [*F*_(1, 7)_ = 0.02, *p* = 0.98]. No influences of trait anxiety on valence and social content processing in the left amygdala were revealed.

No significant effects were found for attachment style measures.

#### Other areas in social brain networks

None of the additional regions of interest activated by social vs. non-social information showed significant effects of trait anxiety or adult attachment scores in the current analyses.

## Discussion

The present fMRI study aimed at investigating relevance detection in the human amygdala by contrasting BOLD signal change in response to visual scenes as a function of both their valence (negative *vs.* positive) and their social (*vs.* non-social) content. Further, we examined the influence of different viewing conditions, implying different emotion regulation strategies, as well as individual differences. This design allowed us to extend previous fMRI studies with a similar purpose (Norris et al., 2004; Britton et al., 2006; Goossens et al., 2009; Scharpf et al., 2010) that did not differentiate between positive vs. negative valence of stimuli—thereby preventing the investigation of any valence × social content interaction effects—and did not take into account task conditions and/or personality traits on relevance processing. Our analysis of amygdala activation revealed a significant interaction between the valence (positive vs. negative) and social content (social vs. non-social) of stimuli. Importantly, these effects were independent of the different viewing conditions (natural viewing, re-appraisal, suppression). Below we discuss these findings each in turn.

### Social content

In whole-brain analyses, bilateral amygdala showed a main effect for social vs. non-social images. This differential social effect was present for neutral as well as emotional stimuli (see Figures 1 and 3A). Such a preferential processing of images according to *social relevance* (Keltner and Kring, 1998; Hariri et al., 2002), even for scenes without any emotional value (“neutral”), converges with previous findings (Norris et al., 2004; Britton et al., 2006; Goossens et al., 2009; Scharpf et al., 2010) and thus extends the notion that the human amygdala is highly sensitive to the social significance of information, presumably due to the intrinsic relevance of social cues for our species.

Importantly, such preferential social vs. non-social processing in our study cannot be attributed to an arousal effect (Anderson et al., 2003; Small et al., 2003), since all social and non-social images were matched on this dimension. These results are consistent with those obtained by (Ewbank et al., 2009) showing that images with “personal impact” activate the amygdala when arousal is controlled for, and may be explained by the fact that social information has an intrinsic motivational value *per se* that may make social images particularly impactful (Morrison and Salzman, 2010). This value, however, is not synonymous with valence (negative or positive), because we found that social relevance and valence were processed interactively within the amygdala with no significant activation difference between positive and negative social images (see below). Consequently, it appears that social relevance is computed in the amygdala independently of the valence and arousal dimensions. This also accords with a key role of this region in processing faces (Todorov et al., 2011; Yang et al., 2012) or gaze (Kawashima et al., 1999; George and Conty, 2008; N'Diaye et al., 2009; Cristinzio et al., 2010), even when face expression is neutral.

It remains, however, to be better determined what are the social cues that preferentially drive amygdala responses, in particular whether they essentially reflect a differential tuning to faces, bodies, and other human features relative to other objects. Selective amygdala activation to faces or gaze has consistently been reported in human and other primate species (Gothard et al., 2007; Hoffman et al., 2007). Such an interpretation would accord with the fact that we observed the same activation pattern reflecting social relevance detection independent of valence in the fusiform gyrus, another brain region known to be preferentially involved in face processing (see also below). This suggests that one possible origin of the social relevance effect could be a more basic reactivity of the amygdala (and fusiform gyrus) toward facial cues or human body parts, (e.g., eyes, etc.) vs. objects or non-human scenes (Vuilleumier et al., 2004). Future studies should therefore address if social significance implied by other visual cues may also trigger a distinctive amygdala response to social relevance.

### Valence

Besides the social relevance effect, we also observed a valence effect in bilateral amygdala, with significantly higher activity during the processing of negative as compared to positive images overall (see Figure 1). This pattern accords with the view that negative information may have an intrinsically stronger relevance for the human organism, probably due to its more immediate implication for survival (Hariri et al., 2002). However, our study demonstrates that this valence effect was predominantly driven by a selective increase to negative information in *non-social* scenes, reflected by a significant valence × social content interaction. The difference between negative and positive social stimuli did not reach significance (see above). Consequently, the notion that negative or threat information has a generally higher value, and is thus linked with distinctive amygdala activation, seems partly inappropriate. According to our data, such “negativity bias” in amygdala responses may arise only (or predominantly) for non-social stimuli—whereas social significance may not need further negative affective significance to be behaviorally relevant. Furthermore, in our data, this valence effect was also likely to be independent of arousal (Morrison and Salzman, 2010), because the most arousing images (negative valence) were associated with heightened amygdala activation only when they were non-social, not otherwise.

### Valence × social content interaction

To our knowledge, by using a full factorial design, our study is the first to report direct evidence for this *interactive processing pattern of valence and social information* in the human amygdala. These results are consistent with the view that the amygdala is critical for relevance detection (Sander et al., 2003, 2005; Adolphs, 2010; Pessoa and Adolphs, 2010), whatever the nature of the relevance, be it social or affective. In future studies, it would therefore be interesting to examine the role of the human amygdala in processing stimuli with different kinds of relevance, including motivational significance (e.g., food or drugs; see Tang et al., 2012, for a recent review).

### Hemispheric lateralization

The valence and social effects, as well as their interaction discussed above, all were found to display a significant hemispheric lateralization in the human amygdala. Whereas emotional images induced more activity in the left than right side in general, both the social *vs.* non-social and the negative *vs.* positive activation differences were stronger in the right than the left side, suggesting a more pronounced general valence × social content interaction in the right amygdala (see Figure 1). In the literature, different hemispheric lateralization accounts of human amygdala function have been proposed, related to language, temporal dynamics, gender (Sergerie et al., 2006), or even awareness (Morris et al., 1998). In our study, lateralization effects were independent of task conditions, suggesting little or no modulation by the degree of covert semantic processing (likely higher during cognitive re-appraisal relative to natural viewing and expressive suppression). Hence, although indirect, this appears inconsistent with the first lateralization account.

On the other hand, the observed activation patterns could be compatible with differential temporal dynamics in amygdala, with possibly quicker habituation effects on the right side. This might result from larger habituation to emotional images in general, and to non-social positive scenes in particular, leading to both the main effect of valence and the valence × social content interaction for the right amygdala. Such habituation would also corroborate the relevance detector hypothesis (Sander et al., 2003, 2005; Adolphs, 2010; Pessoa and Adolphs, 2010) as it might be assumed that the more relevant information should be less prone to habituation, explaining why activity to non-social negative (*vs.* positive) images remained higher in the right amygdala. However, these interpretations remain speculative, as our study did not use direct tests for different lateralization accounts and did not examine differences in the time-course of activation during the experiment. Thus, our findings provide only indirect support to some lateralization in amygdala responses to social and emotional relevance, and more work is needed in the future to better clarify the sources of such asymmetries.

### Personality

Finally, our data also confirms an important role of individual personality differences in modulating amygdala reactivity. In the right amygdala, the valence × social content interaction was only present for low but not high anxious participants (see Figure 4). This was caused by a selective decrease of right amygdala response to social positive (SP) images as a function of trait anxiety. As noted above, this could potentially be attributed to some habituation of the right amygdala to these stimuli that might be further accelerated in high anxious participants because such images were perceived as less relevant to current concerns. This would accord with the negativity biases associated with anxiety and converge with previous findings showing increased processing of (socially) relevant information in the human amygdala as a function of trait anxiety (see Bishop et al., 2004; Sabatinelli et al., 2005; even though such effects were often bilateral in the latter studies). Although an association between STAI-T scores and the interactive response to valence × social content was selectively observed in the right amygdala, a formal ANOVA including SIDE as a factor was not significant. It therefore remains to be determined whether a true lateralization exists regarding personality effects on valence and social content processing in the human amygdala.

### Relevance detection in cortical brain areas

Additional analysis of activation patterns for other areas within the social brain networks (i.e., displaying a main effect of social vs. non-social scenes) also revealed a significant valence × social relevance interaction in right fusiform gyrus (FG), right anterior superior temporal gyrus (aSTG), as well as medial orbito-frontal cortex (mOFC; see Figure 2). Thus, these regions showed a very similar profile of responses as the bilateral amygdala (see above), independent of viewing conditions. However, unlike for the amygdala, we did not find any influence of trait anxiety on right FG, right aSTG, or mOFC.

All of these three regions have previously been associated with privileged processing of social information, including preferential responses to (human) animate stimuli containing faces or bodies in the FG (Kanwisher et al., 1997; Peelen and Downing, 2005; Schwarzlose et al., 2005); representation of abstract social concepts/values and moral sentiments (Zahn et al., 2007), moral cognition (Moll et al., 2005), and social emotion processing (Wicker et al., 2003) in the aSTG; as well as social outcome monitoring (Amodio and Frith, 2006) and theory of mind (Gallagher and Frith, 2003) in the mOFC.

Negative valence effects in FG accord with previous evidence that this area is generally more activated by emotional (*vs.* neutral) and particularly negative (*vs.* positive) information (Vuilleumier et al., 2001, 2004; Hadjikhani and de Gelder, 2003; Surguladze et al., 2003). Effects of valence in aSTG are less clear, because there is not much data regarding emotional and social processing in this area, even though a response to negative information—e.g., fear, anger, and particularly sadness—has often been reported (Levesque et al., 2003; Gillath et al., 2005; Moll et al., 2005). Finally, the current valence effect in mOFC is somewhat at odds with usual considerations that this brain area may primarily be involved in monitoring the positive, rewarding value of social interactions in terms of their probable outcomes (Rolls, 1996; Amodio and Frith, 2006). However, there is evidence that the mOFC is also involved in the attribution of negative value to initially neutral items (Goldstein et al., 2007), which implies that it may have a more general function in computing the subjective value of particularly social stimuli, even if negative, as in our task.

A valence × social content interaction has previously been reported in sensory areas such as the thalamus, superior temporal sulcus and middle occipito-temporal cortex, in addition to the anterior insula and lateral medial prefrontal cortex (Norris et al., 2004; Scharpf et al., 2010), but none of these brain regions were found in the present fMRI experiment. However, as already mentioned above, the emotional relevance effect examined in those studies comprised an emotional *vs.* neutral comparison, but not a 2 (valence) × 2 (social content) interaction, an important difference that is likely to account for the discrepancy between our new and previous findings. Because we looked for brain areas displaying a valence × social content interaction within regions of interest that were selected a priori based on the main effect social vs. non-social, our analysis was more selective. However, using a full 2 (valence) × 2 (social content) experimental design, we did find that valence and social content were processed interactively in several cortical areas including the FG, aSTG, and mOFC (in addition to bilateral amygdala), thus delineating a distributed subcortical-cortical network integrating emotional and social content processing. These regions may constitute a relevance detection network reciprocally interacting with the amygdala, as corroborated by previous findings showing (1) modulation of FG activity by inputs from the amygdala during emotional face perception in humans (Vuilleumier et al., 2001, 2004) and the existence of amygdala-visual cortex projections in macaques (Amaral et al., 2003), (2) simultaneous OFC and temporal cortex activation during social concept representation in humans (Zahn et al., 2007), (3) the presence of direct anatomical connections between the aSTG/middle and inferior temporal lobes and OFC in macaques (Kondo et al., 2003), and (4) functional correlations between amygdala and OFC activity during emotional conflict resolution (Etkin et al., 2006) and the implication of this circuitry in anxiety in humans as well as animals (Bishop, 2007). However, to draw any sound conclusions about causality and directionality of effects within this extended network, more specific investigations of relevance processing in humans within this subcortical-cortical network is necessary.

## Conclusion

The present fMRI study aimed at systematically investigating valence and social content processing within the human amygdala by dissociating between positive and negative, as well as between social and non-social stimuli, in addition to a “neutral” visual baseline. Moreover, we included three different viewing conditions representing “spontaneous” natural emotion processing (natural viewing), as well as cognitive (re-appraisal) and behavioral (expressive suppression) emotion regulation strategies, and also probed for individual differences.

Results revealed an interactive processing of valence and social content in the amygdala, more pronounced on the right than left side. This interaction was not modulated by the different task conditions, but depended on trait anxiety, being significant in low but not high anxious subjects. The latter was due to a selective decrease in amygdala activity to positive social images with higher anxiety scores. Overall, these data suggest that relevance detection in the amygdala operates at a task-independent processing level by integrating both valence and social content. The same valence × social content interaction was present in other cortical regions intimately connected to the amygdala, including the right fusiform gyrus, right anterior superior temporal gyrus, and medial orbito-frontal cortex, suggesting the existence of a distributed subcortical-cortical network for relevance detection in humans.

### Conflict of interest statement

The authors declare that the research was conducted in the absence of any commercial or financial relationships that could be construed as a potential conflict of interest.
